# Elastic wing deformations mitigate flapping asymmetry during manoeuvres in rose chafers (*Protaetia cuprea*)

**DOI:** 10.1242/jeb.225599

**Published:** 2020-12-22

**Authors:** Yonatan Meresman, Gal Ribak

**Affiliations:** 1 School of Zoology, Faculty of Life Sciences, Tel Aviv University, Tel Aviv 6997801, Israel; 2 The Steinhardt Museum of Natural History, Israel National Center for Biodiversity Studies, Tel Aviv 6997801, Israel

**Keywords:** Flexible wings, Flapping flight, Free flight, Insect, Manoeuvring flight

## Abstract

To manoeuvre in air, flying animals produce asymmetric flapping between contralateral wings. Unlike the adjustable vertebrate wings, insect wings lack intrinsic musculature, preventing active control over wing shape during flight. However, the wings elastically deform as a result of aerodynamic and inertial forces generated by the flapping motions. How these elastic deformations vary with flapping kinematics and flight performance in free-flying insects is poorly understood. Using high-speed videography, we measured how contralateral wings elastically deform during free-flight manoeuvring in rose chafer beetles (*Protaetia cuprea*). We found that asymmetric flapping during aerial turns was associated with contralateral differences in chord-wise wing deformations. The highest instantaneous difference in deformation occurred during stroke reversals, resulting from differences in wing rotation timing. Elastic deformation asymmetry was also evident during mid-strokes, where wing compliance increased the angle of attack of both wings, but reduced the asymmetry in the angle of attack between contralateral wings. A biomechanical model revealed that wing compliance can increase the torques generated by each wing, providing higher potential for manoeuvrability, while concomitantly contributing to flight stability by attenuating steering asymmetry. Such stability may be adaptive for insects such as flower chafers that need to perform delicate low-speed landing manoeuvres among vegetation.

## Abstract (precis)

**Summary:** During aerial turning, the chordwise flexibility of rose chafer wings increases the torque of each wing while improving maneouvring stability by mitigating the asymmetry in wing pitch between contralateral wings.

## INTRODUCTION

Locomotion has direct consequences for the evolutionary success of animals. It affects the ability to exploit resources (e.g. food, shelter), find mates, elude predators, migrate and disperse. Consequently, the locomotion of extant species has been shaped by a very long process of natural, and often sexual, selection (Dickinson et al., 2000). The approximately 400 million years of insect flight evolution (Grimaldi and Engel, 2005) has resulted in insects varying not only in the size and shape of their wings but also in the number of wings used for flight and the internal structure of the wing veins that give the wing its shape and rigidity (Wootton, 1981).

Because of their smaller size and different flapping kinematics, insects engage with the surrounding air in a different manner to that of vertebrates (Chin and Lentink, 2016). At Reynolds numbers of ∼10^2^–10^4^, typical of most insects, the relative contribution of air viscosity to flow patterns increases, affecting the attachment of airflow to the wings and, consequently, the aerodynamic forces generated by these wings (Dudley, 2000). In addition, vertebrate wings can be actively shaped, using muscles and skeletal joints, to modulate the aerodynamic and inertial forces required for aerial manoeuvres and perturbation recovery (Hedenström and Johansson, 2015; Lentink et al., 2007). Insect wings, in contrast, lack intrinsic musculature, thereby restricting their active control over wing shape during flight. However, insect wings elastically deform during flapping, resulting in wing twist and camber – two important features that can affect the wing's efficiency in producing aerodynamic forces (Ennos, 1988; Wootton, 1992; Young et al., 2009).

Wing twist can compensate for the span-wise increase in the angle of attack (AoA) as a result of flapping during forward flight (Pringle, 1957; Walker et al., 2009; Wootton, 1981; Young et al., 2009). Unlike in most flying vertebrates, insect wings twist in both directions, ensuring lift production during both the upstrokes and downstrokes (Sane, 2003; Wootton, 1981). Wing camber improves flow attachment to the wing, increases the lift and delays flow separation during dynamic stall and stroke reversals, where the AoA reaches ±90 deg (Shyy et al., 2016; Wang et al., 2003). Therefore, it is generally accepted that some degree of wing flexibility is required in insects. However, there is controversy as to whether wing deformations have an advantage over rigid wings in aerodynamic force production. Whereas studies have shown that wing flexibility enhances load-lifting capacity (Mountcastle and Combes, 2013), down-wash and lift production (Du and Sun, 2010; Nakata and Liu, 2012; Shoele and Zhu, 2013), delays stall during the translational phase (Lehmann, 2004; Marden, 1987), improves wake capture (Vanella et al., 2009) and flight efficiency (Jankauski et al., 2018; Young et al., 2009; but see Lehmann et al., 2011, for energy losses associated with flexible wings) and increases tolerance to aerial perturbations (Mistick et al., 2016), other studies have suggested that rigid wings would produce greater lift (Tanaka et al., 2011; Tobing et al., 2017; Zhao et al., 2010). These different, sometimes conflicting, findings may arise from the use of simplified model wings and numerical simulations, whereas empirical measurements of real insect wing deformation during flight are scarce. Consequently, measuring wing deformation during free flight is crucial for a deeper understanding of the intricate fluid–structure interaction that underlies the benefits and disadvantages of flexible wings.

Although most manoeuvres originate from asymmetric force production by contralateral wings, the flapping kinematics by which insects produce the asymmetric force are not restricted to a single, uniform mechanism. Different steering kinematics have been shown in Odonata (Li and Dong, 2017), Hymenoptera (Vance et al., 2013), Lepidoptera (Greeter and Hedrick, 2016) and Diptera (Bergou et al., 2010; Dickinson and Muijres, 2016; Vance et al., 2013). Aerial manoeuvres can result from bilateral asymmetries between the wings' stroke planes, flapping amplitudes (within the stroke plane), deviation of the wing's trajectory out of the stroke plane (deviation angle), and in wing pitch (the angle of wing rotation about its length) (Bergou et al., 2010; Dickinson and Muijres, 2016; Li and Dong, 2017). However, studies reporting steering kinematics have too often ignored elastic wing compliance during steering, which could potentially affect the manoeuvre dynamics.

**List of symbols and abbreviations**

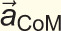
instantaneous wing acceleration at its centre of mass
AA: analis anterior vein
AoA: angle of attack (α)
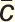
mean chord length
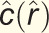
non-dimensional chord length
*C*_D_: drag coefficient
*C*_L_: lift coefficient
*C*_rot_: theoretical rotational force coefficient
CoM: centre of mass
CuA: cubitus anterior vein
*e_x_*_,*y*,*z*_: *x*, *y* and *z* components of the rotation axis, respectively, in the body frame of reference.
*F*_a_: aerodynamic inertial force
*F*_rot_: rotational force
*F*_trans_: translational force
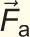
instantaneous inertial force due to the added mass
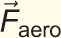
total instantaneous aerodynamic force
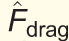
direction of drag force (unit vector)
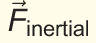
instantaneous inertial force emerging from acceleration of wing mass
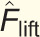
direction of lift force (unit vector)
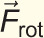
instantaneous rotational force
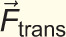
instantaneous translational force
*I*: moment of inertia
*m*_w_: wing mass
*M*_actual_: torque estimated from body rotation
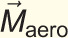
instantaneous aerodynamic force moment (aerodynamic torque)
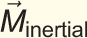
instantaneous inertial torque
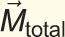
instantaneous torque generated by each wing
mj: marginal joint
MP: media posterior vein
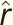
non-dimensional radial position along the wing length
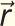
instantaneous moment arm of 0.725 wing length
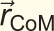
moment arm of 0.29 wing length
RP: radius posterior vein
*S*: wing area
SPA: stroke plane angle
*U*_t_: wing tip speed
wb: wing base
wt: wing tip
*x*, *y*, *z*: longitudinal, lateral, and dorso-ventral body axes
*Y_w_*: axis parallel to the wing chord
*Z_w_*: normal to the leading edge plane
α_f_: incidence angle for the compliant wing
α_r_: incidence angle for the rigid wing
β: stroke plane angle
ρ: air density
θ: incidence angle
ϕ: flapping angle
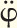
angular acceleration
ω: angular velocity for rotation about the wing length

**Fig. 1.**
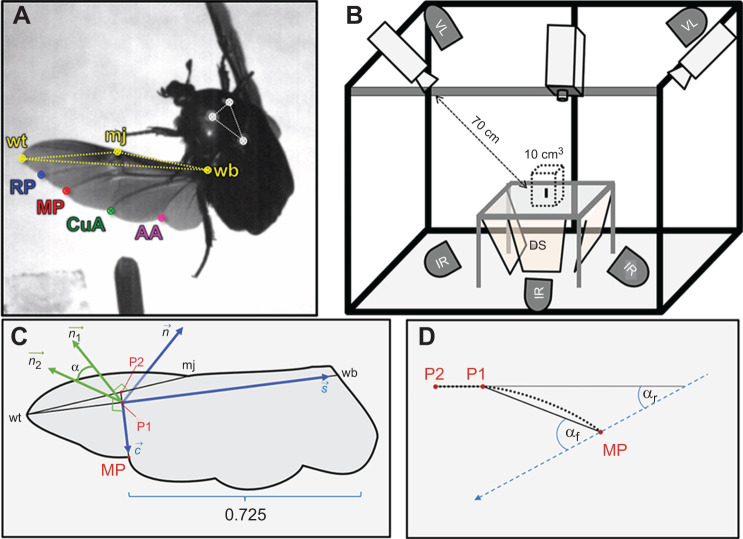
**Methodology.** (A) A free-flying *Protaetia cuprea* and the digitized landmarks. The three white dots on the thorax were used to extract body orientation. Colour-coded dots denote wing landmarks used to extract flapping kinematics and wing deflection at the trailing edge: wb, wing base; wt, wing tip; mj, marginal joint; AA, analis anterior vein; CuA, cubitus anterior vein; RP, radius posterior vein; MP, media posterior vein (terminology follows Haas and Beutel, 2001). Triangles illustrate the planes on the body and wing that were calculated using the position of the three landmarks. (B) Schematic illustration (not to scale) of the experimental filming set-up. Three high-speed cameras were mounted above a calibrated mutual field of view to capture the flight of the beetle post take-off. IR, infra-red floodlights; DS, diffusive screens; VL, visible floodlights. (C) Schematic planform and (D) profile views of the points on the wings used for calculating the angles of incidence relative to the stroke plane. Point *P*1 is at 0.725 the distance between the wing base and wing tip. Point *P*2 is located at the intersection point between instantaneous chord length 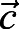 and the mj–wt vector. It is located on the wing chord at 0.725 of the wing length as well. C also shows an illustration of the velocity, span and chord vectors (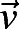, 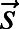 and 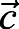, respectively) measured from the digitalized wing and the 
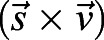
 and 
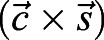
 cross-products (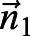 and 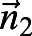, respectively). α is the angle between 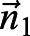
and 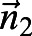, and is equal to the angle of attack (AoA). Red points illustrate the landmarks used for calculation. The dashed black line represents the profile of the thin flexible wing. The dashed blue line represents a hypothetical stroke plane with an arrow pointing towards the direction of the wing's translation. Solid black and grey lines (in D) illustrate the angle of incidence calculated for the flexible (α_f_) and rigid (α_r_) wings. A and B were adopted from Meresman and Ribak (2017).

Here, we focused on the rose chafer (*Protaetia cuprea*; Fig. 1A) and analysed how wing flexibility affects turning dynamics during flapping flight. Rose chafers are pollen feeders, frequently manoeuvring to land in complex 3D environments. During low-speed flight, their wings undergo extensive elastic chord-wise deformations, particularly in the proximal section of the trailing edge, resulting in the formation of considerable twist and positive camber (Meresman and Ribak, 2017). We asked how these extensive wing deformations relate to this beetle's ability to produce low-speed manoeuvring flight. Wing deformation may have a damping effect on the asymmetric flapping of contralateral wings, helping to restore stability; or it may promote aerial instability and consequently allow sharper manoeuvring. To determine which of these possibilities is correct, we measured wing deformation in free-flying rose chafers performing low-speed aerial manoeuvres. Using high-speed video cameras, we measured wing kinematics and deformation of the left and right wings and compared these deformations between the contralateral wings. To complement the measurement of wing kinematics, we performed a blade-element analysis to estimate the effect of wing compliance on the turning torque produced by the wings.

## MATERIALS AND METHODS

### Insects

Adult rose chafers, *Protaetia cuprea* (Fabricius 1775), were obtained from a laboratory population, established in 2014 from insects collected in the field. Wild adults are added annually to prevent a founder bottleneck effect. The beetles were fed apples and provided with compost based on oak leaves for burrowing, oviposition and development of larvae. Males and females have no apparent inter-sex difference in morphology or behaviour (Meresman and Ribak, 2017). Therefore, we pooled sexes in the experiments.

### Filming procedure

The beetles were marked with three dots on the pronotum (Fig. 1A), using acrylic paint or adhesive paper, and their body mass was measured to the nearest 0.1 mg using an analytical balance (BAS 32 plus, Boeco, Hamburg, Germany). Both marking methods resulted in negligible added mass (<1 mg) and did not seem to affect the beetles' behaviour or flight performance. The marked beetles were placed individually on top of a vertical 4 cm rod in the centre of an open flight arena. Three synchronized high-speed cameras (Fastcam SA3_120K, Photron Inc., San Diego, CA, USA) were fitted with 50 mm lenses and set to film at 3000 frames s^−1^ (shutter speed 66.7 μs, resolution: 768×768 pixels). We placed three LED infrared projectors pointing at each camera to provide background illumination and two 500 W visible light projectors were mounted from above (Fig. 1B). To calibrate the cameras spatially, either a 10 cm^3^ calibration cube or an 8 cm long wand were used with the software DLTcal5 and easywand5, respectively (Hedrick, 2008; Theriault et al., 2014).

In the flight arena, we allowed the beetles to take off undisturbed in stagnant air multiple times. Each beetle was filmed until it either was reluctant to continue flying or had completed 10 flights. Of the 46 beetles that flew, only 23 performed manoeuvres within the spatially calibrated area. From each of these 23, we selected the film in which it had performed the largest rotation (in degrees) about its longitudinal body axis within three flapping cycles (approximately 30 ms; Movie 1).

### Data analysis

#### High-speed film analysis

From each frame in each film, we digitalized 17 landmarks: the three pronotum points described above and seven natural landmarks on each wing: wing base (wb), marginal joint (mj) and wing-tip (wt) on the leading edge and the tips of the analis-anterior (AA), cubitus-anterior (CuA), media-posterior (MP) and radius posterior (RP) on the trailing edge (see Haas and Beutel, 2001, for terminology and Fig. 1A for actual landmark locations). We used the digitized landmarks to track both position and orientation of the beetles during flight and to extract flight speed, flapping kinematics and wing deformations. The methodology was previously described (Meresman and Ribak, 2017). Therefore, part of the description is repeated below verbatim (see Harriman and Patel, 2014) for easy access.

#### Flight speed and acceleration

From the instantaneous position of the marked scutellum (posterior white point in Fig. 1A), in each video frame we found the instantaneous flight velocity and mean acceleration of the beetles by fitting the time series of instantaneous positions with a second-order polynomial function, and finding its first and second time derivatives. The mean *R*^2^ (±s.e.m., *n*=23) values for the polynomial curve fitting functions of the time series of position data on the *X-*, *Y-* and *Z*-axes were 0.994±0.006, 0.991±0.013 and 0.852±0.208, respectively.

#### Normalizing the flapping cycle duration

Each analysed film section comprised three flapping cycles. To control for variance in cycle duration, we divided the serial numbers of each film frame by the number of frames within a flapping cycle, to give a non-dimensional time scale of flapping cycles in the range 0–3.

#### Instantaneous body orientation and body frame of reference

We used the three marked points on the thorax (Fig. 1A) to define a body-fixed Cartesian coordinate system, in each frame, in which the longitudinal body axis (*x*) is represented by a vector connecting the scutellum with a point half-way between the other two points on the pronotum. The lateral body axis (*y*) was defined as the vector connecting the right and left points on the pronotum. The dorso-ventral body axis (*z*) was found from the cross-product:(1)
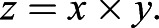
The origin was defined between the wing bases, and all body axes vectors were converted to a unit vector. We used the body axes as a rotation matrix to: (1) transform all instantaneous landmark positions on the wing to a body frame of reference; and (2) define the turning of each beetle using the Euler axis–angle convention.

For the former, the origin was shifted to the relevant wing base and the vector cosines of the three body axes were used as a rotation matrix to convert each wing position to the body frame of reference (*x*,*y*,*z*). Thus, any position on the wing in the camera frame of reference *P*(*X*,*Y*,*Z*) is rotated to *P*′(*x*,*y*,*z*) in the body frame of reference according to:(2)
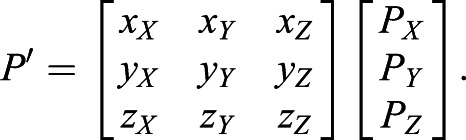
The procedure was repeated for each frame, allowing extraction of the flapping kinematics of the wing in a frame of reference that moves with the rigid body.

#### Quantifying the turns in air

**Fig. 2.**
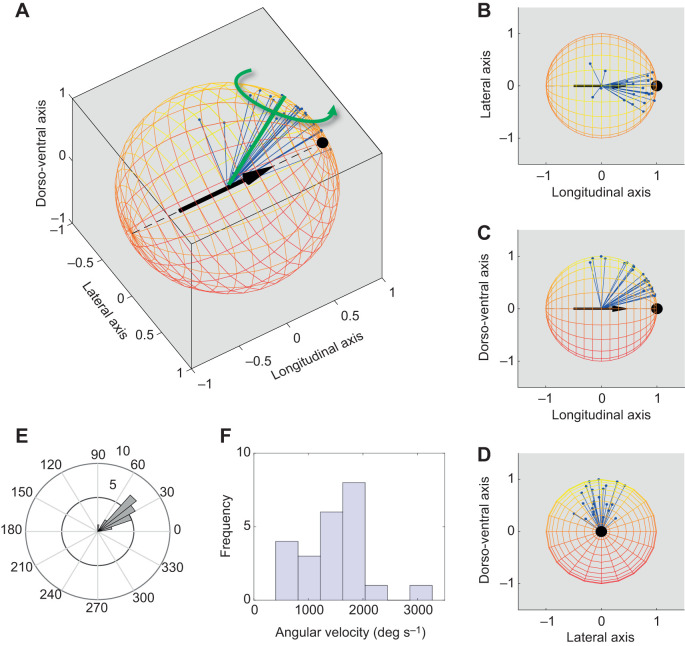
**Body rotation of the free-flying beetles.** (A) 3D visualization of the Euler axis rotations. Here, all rotations are displayed together as rotations towards the left of the beetle by inverting (mirror image) all the rotations to the right. The black arrow marks the longitudinal axis of the beetles, with the head pointing towards the pole of the sphere marked by a black dot. Blue lines represent the rotation axes of the 23 beetles. The green line and arrow represent the mean rotation axis and the direction of the rotation, respectively. (B–D) Same as in A, but illustrating the rotation axes relative to the different body axes. (E) Angular histogram showing the distribution of rotation magnitudes about the rotation axes. (F) Histogram of mean angular velocity for the same data. Arrows in A–D were produced using the Matlab-compatible function mArrow3 (© 2009, Georg Stillfried; all rights reserved).

The mean turn rate, turn magnitude and axis for the manoeuvring beetles were found from the beetles' body orientation in the camera frame of reference using the Euler axis–angle representation (Fig. 2). In this representation, the change in orientation of a rigid body can be described by a vector in 3D space that depicts the rotation axis, and by the angle that depicts the rotation about the axis according to the right-hand rule. We used the three body axes (in the camera frame of reference) in the first and last frames of the films showing the manoeuvre, and rotated the body axes at the end of the turn into the beetle's frame of reference before the turn:(3)
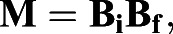
where **B**_i_ and **B**_f_ are 3×3 matrices representing the direction cosines of the three body axes in the camera frame of reference, i.e.
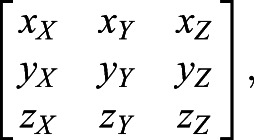
at the beginning and end of the turn, respectively. The Euler axis and angle can be extracted directly from the components of the 3×3 rotation matrix **M** as angle θ:(4)
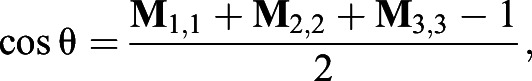
axis *e*:(5a)
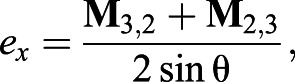
(5b)
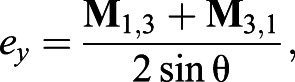
(5c)
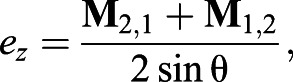
where *e_x_*, *e_y_* and *e_z_* are the *x*, *y* and *z* components of the rotation axis, respectively, in the body frame of reference.

#### Wing flapping kinematics

Wing flapping kinematics was extracted as three time-varying angles (flapping, deviation and incidence) as in Meresman and Ribak (2017), but with the incidence angle being calculated twice: once as the wing pitch relative to the stroke plane for the flexible wing area (α_f_) near the trailing edge; and again for the rigid wing area (α_r_) near the leading edge (Fig. 1C,D). The instantaneous wing tip data in the body frame of reference and in the sagittal plane wt(*x*,*z*) were used to find the stroke plane of each wing from the linear least square error line of these positions throughout the three flapping cycles. The stroke-plane angle (SPA) is defined as the slope of the line as in Ellington (1984a), but in the body frame of reference.

**Fig. 3.**
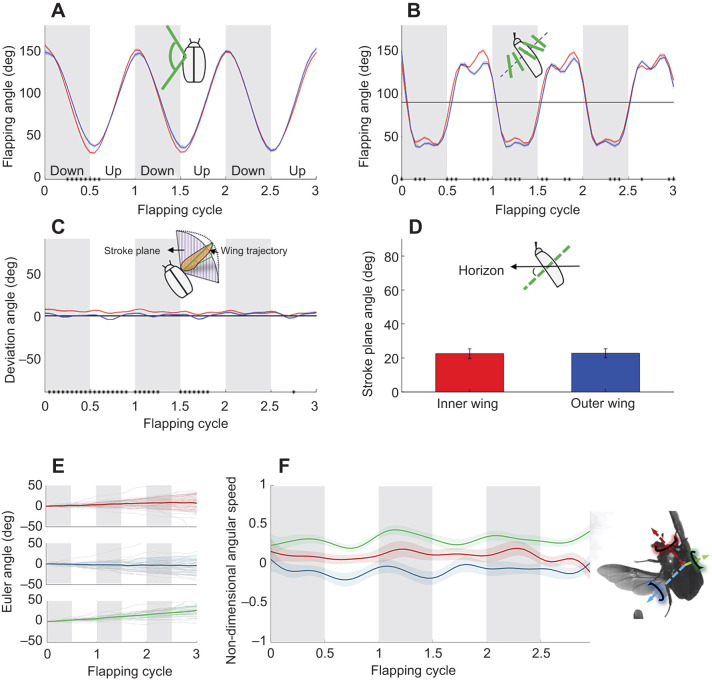
**Wing kinematics.** Mean (±s.e.m., *n*=23) (A) flapping, (B) incidence, (C) deviation, (D) stroke-plane angle (see insets with schematic illustrations for definitions) and (E) time-varying Euler angles during three flapping cycles of free-flying *P. cuprea*, performing hovering aerial manoeuvres. The inner wing (e.g. left wing in a left turn) is marked in red and the outer wing in blue. Grey areas denote the downstrokes. The standard error is denoted by the lighter colours in A–C and E–F and by the error bars in D. Black dots on the horizontal axes mark significant statistical differences between the inner and outer wings in the instantaneous angles (two-tailed, paired *t*-tests, *P*<0.05). (F) Change in the non-dimensional angular speed during the three flapping cycles comprising the manoeuvre. Non-dimensional angular speed is the instantaneous angular speed divided by the maximal instantaneous angular speed for each manoeuvre. Red, blue and green colours in E and F denote roll pitch and yaw of the beetles' body, respectively, as illustrated in the inset on the right.

Next, we rotated wing data by SPA about the *y*-axis. The instantaneous flapping and deviation angles are defined as the azimuth and elevation of the wing length relative to the stroke plane. Wing incidence was defined as the rotation of the wing about its length (see Fig. 3B). To find this angle in each film frame, for the flexible and rigid wing parts, we rotated the wing about the *z*- and *x*-axes so that the span aligned with the *y*-axis. We then found the vectors connecting MP (Fig. 1A) with point *P*1, and *P*1 with *P*2, where all three points are located on the wing chord at 0.725 wing length (Fig. 1C). The angle between each of these vectors and the stroke plane in the *xz* plane was the instantaneous incidence angle for the flexible (α_f_) and the rigid (α_r_) wing area, respectively. We chose to measure the incidence angle using MP at 0.725 wing length because of the expected proximity of this spanwise position to the centre of pressure of a flapping wing (∼0.7 in fruit flies; Fry et al., 2005).

#### Chord-wise wing deformations

Wing deformation was measured relative to the leading edge. Using the three leading edge landmarks (wb, mj and wt; Fig. 1A), we defined an instantaneous plane in 3D space that represents the (relatively) rigid surface of the wing. Next, for the four landmarks on the trailing edge (RP, MP, CuA and AA; Fig. 1A) we calculated their perpendicular distance from this plane. These perpendicular distances represent the chord-wise deflections at these positions along the wing. Deflections of the trailing edge points defined the local wing camber whereas the pattern of these deflections along the wing length gave the wing twist.

To measure the rigid wing plane and deflection of trailing edge landmarks from the digitized data, we first shifted the origin of the body fixed coordinate system to the wing base (point wb in Fig. 1A). We then defined two vectors connecting wb with two non-colinear points on the leading edge (mj and wt in Fig. 1A). The cross-product of the two vectors defines a vector (*Z*_w_) normal to the plane formed by the three points (yellow triangle in Fig. 1A):(6)
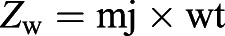
and(7)
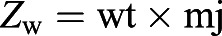
for the left and right wings, respectively.

The direction of the wing chord (*Y*_w_) is defined as the cross-product of *Z*_w_ and wt:(8)
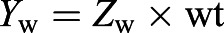
and(9)
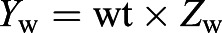
for the left and right wings, respectively, where all vectors are converted to unit vectors.

Using the vector cosines of the three wing axes (wt, *Y*_w_, *Z*_w_) as a rotation matrix, we transformed all data points on the wing to the wing frame of reference defined by the leading edge. The transformation of a given point *P* from the frame of reference of the camera to the frame of reference of the leading edge (*P′*) is:(10)
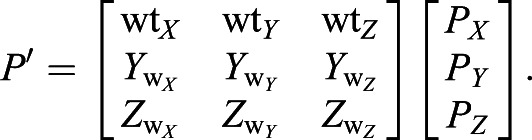
The procedure was repeated for each film frame. By definition, the *Z*_w_ component of the transformed data points is the deflection of this point out of the plane of the rigid leading edge.

### Net turning moment estimation

We estimated the instantaneous forces that emerge from flapping the contralateral wings based on the wingtip velocity and the AoA of the wings. We then used the forces of the left and right wings to estimate the net turning torque generated by the asymmetric flapping. The calculations were performed once for flexible wings using the AoA of the flexible wing area (α_f_) and a second time for ‘rigid’ wings using the AoA for the rigid wing area (α_r_).

#### Calculation of the AoA

To find the AoA for the left wing we defined 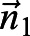 and 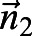 (Fig. 1C) as the cross-products:(11)
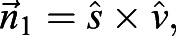
(12)
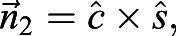
where 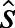, 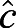 and 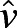 are unit vector in the direction of *P*1–wb, *P*1–MP and the velocity of the wing relative to air at *P*1, respectively, as shown in Fig. 1C. The instantaneous velocity was found numerically from the position of *P*1 as in Rayner and Aldridge (1985). The geometric AoA was taken as the angle between 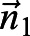 and 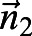 using the dot product:(13)
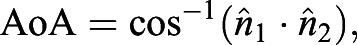
where 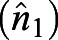 and 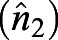 are the unit vectors of 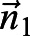 and 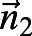, respectively. For the right wing, the procedure is the same, but with Eqns 11 and 12 adjusted according to the ‘right-hand rule’. Next, we repeated the procedure to calculate the AoA for the rigid (leading edge) part of the wing using the vector connecting *P*2 with *P*1 instead of P1 and MP (Fig. 1C).

#### Estimation of the aerodynamic force

The instantaneous quasi-steady aerodynamic translational force (*F*_trans_) is (Sane and Dickinson, 2002):(14)
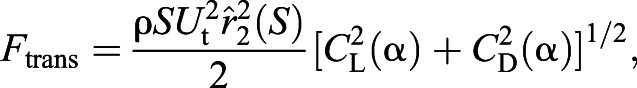
where ρ is the air density (taken as 1.225 kg m^−3^), *S* is wing area, *U*_t_ is the wingtip speed, 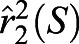 is the non-dimensional second moment of area, and *C*_L_ and *C*_D_ are the lift and drag coefficients, respectively. The instantaneous lift and drag coefficients were estimated as a function of the wing's instantaneous AoA as in Meresman and Ribak (2017) assuming:(15)
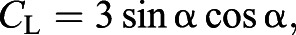
(16)
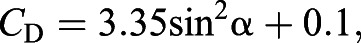
where α is the AoA.

The rotational force (*F*_rot_) is defined as (Sane and Dickinson, 2002):(17)
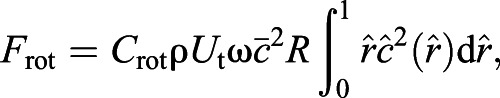
where *C*_rot_ is the theoretical rotational force coefficient. For lack of a better estimate, we assumed a rotation axis at 0.25 the wing chord from the leading edge. The position of this axis on insect wings is typically at 0.25–0.5 of the wing chord (Ellington, 1984b). Thus, our choice giving *C*_rot_=1.57 (Sane and Dickinson, 2002) uses the upper value of this coefficient. ω is the angular velocity for rotation about the wing length, 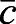 is the mean chord length, 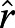 is the non-dimensional radial position along the wing length and 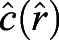 is the non-dimensional chord length.

The aerodynamic inertial force (*F*_a_) is (Sane and Dickinson, 2002):(18)
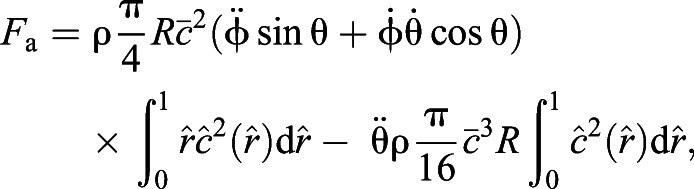
where φ is the flapping angle and θ is the incidence angle. The incidence angle is calculated relative to the stroke plane; thus, we used this angle as the AoA for the estimation of *F*_a_. To find the time derivative of these angles, we first smoothed the data with a low-pass Butterworth filter with a cut-off frequency of 300 Hz (∼3 times the flapping frequency), then calculated the derivatives numerically as in Rayner and Aldridge (1985).

Because the deviation angle was negligible (Fig. 3) the instantaneous directions of the lift and drag vectors were found in the body frame of reference using the stroke plane angles, assuming drag is always parallel to the stroke plane and lift is perpendicular to it. We calculated separately the magnitudes of lift and the drag in Eqn 14 and determined their direction according to the SPA (stroke plane angle β, see Fig. S1) as:(19)
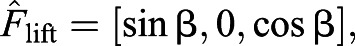
(20hboxa)
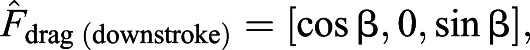
(20hboxb)
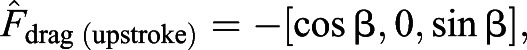
where 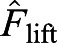 and 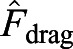 are the directions of the forces (unit vectors).

Vector summation of the lift and drag for each wing gave our estimate of the instantaneous translational force:(21)
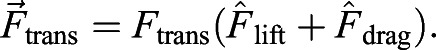
To calculate the direction of *F*_rot_ and *F*_a_, we summed their magnitudes and multiplied them by a unit vector normal to the wing area (Sane and Dickinson, 2002). Finally, vector summation gave the total instantaneous aerodynamic force as:(22)
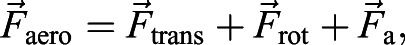
where 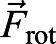 and 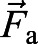 are instantaneous rotational and inertial force, respectively.

#### Estimation of wing inertia

To the aerodynamic forces we added the instantaneous inertial force 
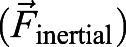
 due to the accelerating mass of the wing. We estimated the wings' centre of mass (CoM) to be at 0.29 the distance between the wing base and the wing tip based on total wing mass and mass distribution of five *P. cuprea* wings reported elsewhere (Meresman and Ribak, 2017). The position of the CoM was used to find the instantaneous acceleration of the wing from its second time derivative of the position data 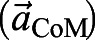. The instantaneous inertial force of the wing mass is represented as:(23)
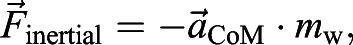
where *m*_w_ is the total wing mass.

#### Calculation of the quasi-steady torque

**Fig. 4.**
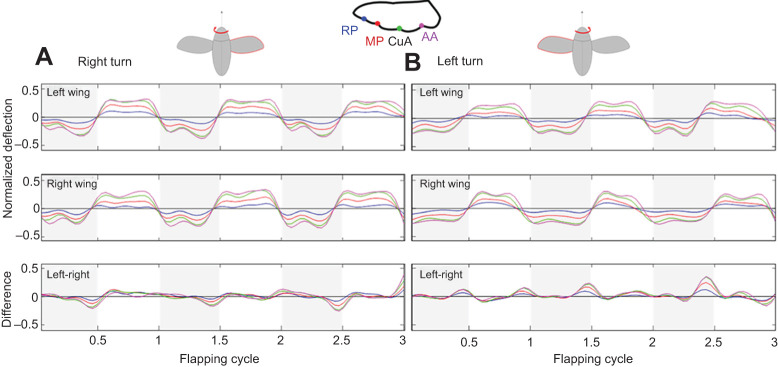
**Wing deformation.** Examples of trailing-edge deflections taken from two individual rose chafers during aerial manoeuvres where the beetle is turning towards its right (A) and its left (B). The top and central panels show the deflection from the left and right wings, respectively. The bottom panel shows the difference in the deflections (left minus right wing). Colours correspond to colour coded landmarks in Fig. 1A. The ‘inner wing’ (i.e. left wing in a turn to the left of the beetle and right wing in a turn to the right) is highlighted in red in the insets representing the turning beetles (top).

Using the instantaneous moment arm between the hinge and *P*1 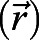, the instantaneous aerodynamic force moment of each wing is:(24)
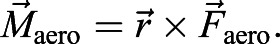
For the force moment due to inertia of the wing mass we rotated 
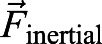
 and the moment arm from the wing hinge to the wing CoM 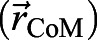 to the beetle's body frame of reference and calculated the instantaneous inertial torque as:(25)
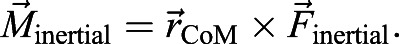
The total instantaneous torque per wing is the summation:(26)
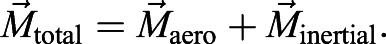
The torques from the inner wing and the outer wing (see inset in Fig. 4 for definition of ‘inner’ and ‘outer’ wings) were summed, to give the net instantaneous torque acting on the beetles' body:(27)
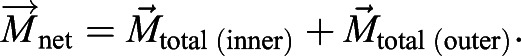
The vector representation of the net torque was broken into its *x*, *y* and *z* components to give the torques about these body axes.

### Torque estimation from body rotation

We compared the obtained magnitude and direction of net torque with an estimate of the torque (*M*_actual_) required to achieve the body rotations observed in the films. The three Euler angles of the body were extracted from the direction cosine matrix of the body axes in the camera frame of reference using the Matlab function dcm2angle. The second derivative of the Euler angles (i.e. the angular acceleration) was calculated, rotated to the beetle's body orientation and multiplied by the mass moment of inertia tensor of a prolate spheroid according to the following equation:(28)
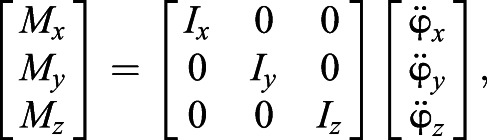
where *I_x_*, *I_y_* and *I_z_* are the moment of inertia about the primary axes and 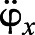, 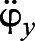 and 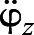 are the angular acceleration about these axes (roll, pitch and yaw, respectively; Fig. S2). Note that the estimation ignores any coupling (Coriolis forces) and does not include damping from air resistance, which should somewhat increase the torque at turn initiation and reduce it at turn termination.

We calculated the moment of inertia for the body as a prolate spheroid using the mean mass (*m*=6.9×10^−4^ kg), body length (*H*=0.0208 m), width (*W*=0.0128 m) and depth (*D*=0.0081 m) of *P. cuprea*.

The major axis (*a*) was the beetle's length (*H*). The minor axis of the spheroid (*b*) was defined as the mean of the maximal and minimal cross-section:(29)
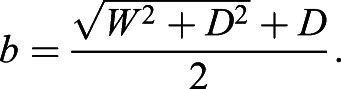
The moment of inertia of the prolate spheroid about the primary *x*-, *y*- and *z*-axes is:(30)
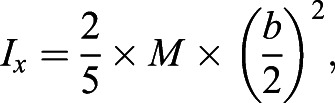
(31)
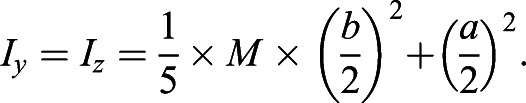


### Statistical analyses

Wing kinematics and deformation during the three flapping cycles of the 23 beetles were averaged every 5% of the flapping cycle duration by interpolating the data into 60 non-dimensional time steps using cubic-spline. The turns analysed were to the left or right side of the beetle. We therefore use the term ‘inner’ and ‘outer’ wings (Fig. 4) where the inner wing is the left wing in a turn towards the left of the beetle and the right wing when the beetle is turning to the right. Paired *t*-tests were performed to test for differences in instantaneous wing kinematics between the inner wing and the outer wing throughout the flapping cycles. Data are reported as means±s.e.m.

## RESULTS

### Manoeuvring dynamics and wing kinematics

The beetles in the films (*n*=23) demonstrated a mean flight speed of 0.41±0.18 m s^−1^ and a mean wingbeat frequency of 110±2 Hz. The average wing length was 2.08±0.05 cm. The mean wingtip velocity due to flapping (9.13±0.19 m s^−1^) was much higher than the beetles' (3D) flight speed, giving an advance ratio (flight speed/wingtip speed=0.045±0.003). This advance ratio is low enough to assume hovering conditions (Ellington, 1984a). Similarly, the vertical component of flight speed was only 0.1±0.04 m s^−1^; therefore, we ignored any vertical inflow. The beetles rotated about the rotation axis by 39±3 deg on average and at a mean turn rate of 1429±124 deg s^−1^ (Fig. 2). Fig. 3E,F illustrates the change in the beetles' Euler angles and their time derivatives (angular speeds) during the three flapping cycles comprising the manoeuvre.

During turning, the wingbeat amplitude of the inner wing was higher than that of the contralateral wing (Fig. 3A). It also slightly preceded the outer wing in stroke reversals (Fig. 3A,B). Differences in flapping kinematics were pronounced during the first flapping cycle but began to subside in the second flapping cycle, and by the beginning of the third flapping cycle there was no apparent difference in wing flapping angle. Moreover, after the third wing supination (ventral stroke reversal), the flapping asymmetry seemed to reverse direction, with the outer wing leading and having higher amplitude (Fig. 3). The incidence angle (α_f_) demonstrated a similar waning pattern (between flapping cycles 1–3) to that of the flapping angle. At mid-downstroke, the incidence angles were slightly larger in the inner wing (Fig. 3; Table S1). At mid-upstroke, the inner wing performed smaller wing pitch relative to the direction of wing movement (Fig. 3B). The maximal contralateral difference in incidence angle was higher at the upstroke (19.1 deg at 0.85 of the flapping cycle) than at the downstroke (8.22 deg at 0.2 of the flapping cycle). The deviation angle slightly differed between the wings, with the inner wing deviation angle being higher (Fig. 3C; Table S1). The stroke plane angle remained similar between the wings (paired *t*-test, *t*_22_=0.528, *P*=0.603; Fig. 3).

### Wing deformation

The wings of *P. cuprea* elastically deformed during flapping. During the downstroke, the trailing edge deflected towards the ventral side of the wing, forming wing twist and camber (Fig. 4). During the upstroke, the trailing edge deflected towards the dorsal side of the wing, restoring wing twist and camber in the inverted wing. While the entire trailing edge elastically deformed throughout the flapping cycle, the magnitude of deflection decreased gradually from wing base to wing tip (Table 1, Fig. 4). Turning resulted in consistent contralateral asymmetry in elastic wing deformation (Fig. 4).

**Table 1.**
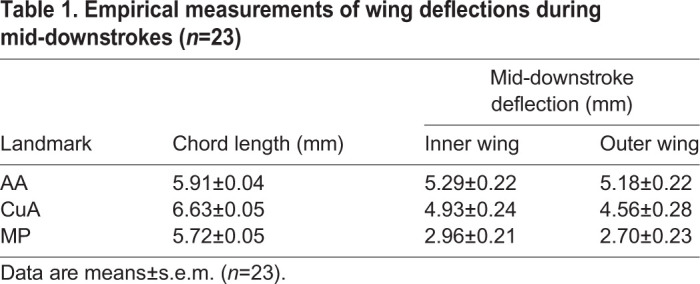
**Empirical measurements of wing deflections during mid-downstrokes (*n*=23)**

**Fig. 5.**
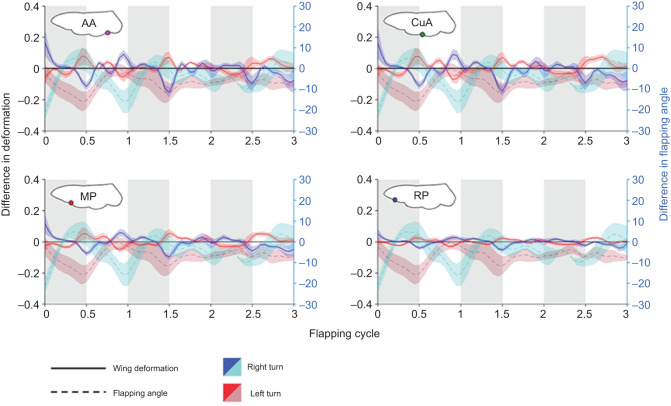
**Instantaneous** **difference in wing kinematics and deformation.** All differences are left minus right wings; lines are means and shaded areas are s.e.m. Differences in left (*n*=13) and right (*n*=10) manoeuvres are denoted in warm and cold colour palettes, respectively. Kinematic differences are denoted in dashed maroon and cyan lines. Deformation differences are denoted in blue and red. Grey areas denote downstroke phase.

**Fig. 6.**
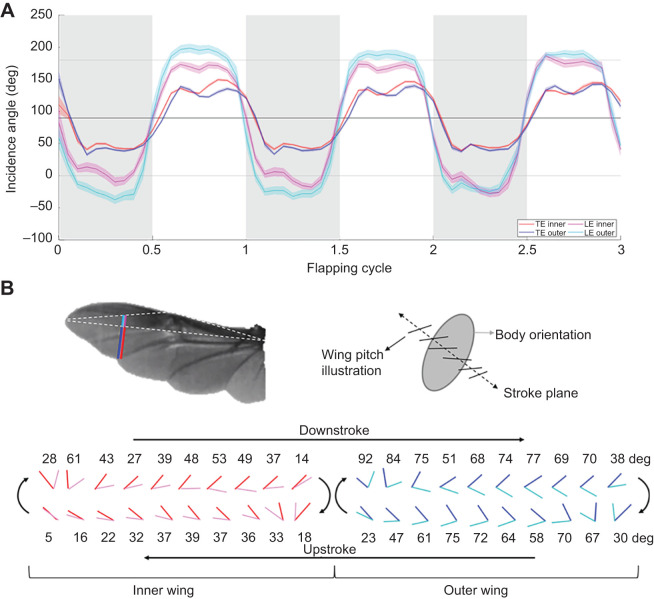
**Effect of wing compliance on incidence angle.** (A) Instantaneous incidence angle during three flapping cycles. Means (lines) and s.e.m. (shaded area) for 23 beetles. Grey areas denote downstrokes. Inner and outer wings are denoted in warm and cold colour palettes. Red and blue lines denote incidence angles calculated from the trailing edge to the rigid leading edge plane at 0.725 of wing length. Cyan and magenta lines denote the incidence angles of the leading edge (rigid) plane. Note that the incidence angle is defined relative to the anterior side of the stroke plane, i.e. the angles are >90 deg during the upstroke and 90 deg implies that the wing is moving perpendicular to its area. (B) Time series showing the difference in the incidence angle during the first flapping cycle. Numbers denote the difference in the incidence angle between rigid and flexible wings. The incidence is calculated relative to the stroke plane (see schematic illustration). At the bottom, the lines representing rigid and flexible parts of the inner and outer wings converge at the trailing edge side and the wing moves to the right and left in the downstroke and upstroke phases, respectively.

The highest instantaneous differences in wing deformation between the contralateral wings occurred at stroke reversals, during which differences in the flapping angles were also highest. Smaller but obvious asymmetries also occurred during midstroke (Fig. 5). The contralateral asymmetry in wing deformation was coupled with the manoeuvre direction, i.e. when turning to the right, the trailing edge of the right wing experienced greater deformation than the trailing edge of the left wing, and vice versa. The angle of incidence for the compliant wing (α_f_), shown in Fig. 3B, was larger than the angle of incidence that the wings would have achieved had they been rigid (α_r_). The trailing edge deflections increased the incidence angle on both the downstrokes and upstrokes and had an attenuating and even reversing (in the upstroke) effect on the bilateral asymmetry in the incidence angle of the rigid leading edge. Moreover, the elastic deformation appears to have had an attenuating effect on the rate of incidence angle change during stroke reversals (Fig. 6).

### Estimated forces and torques

**Fig. 7.**
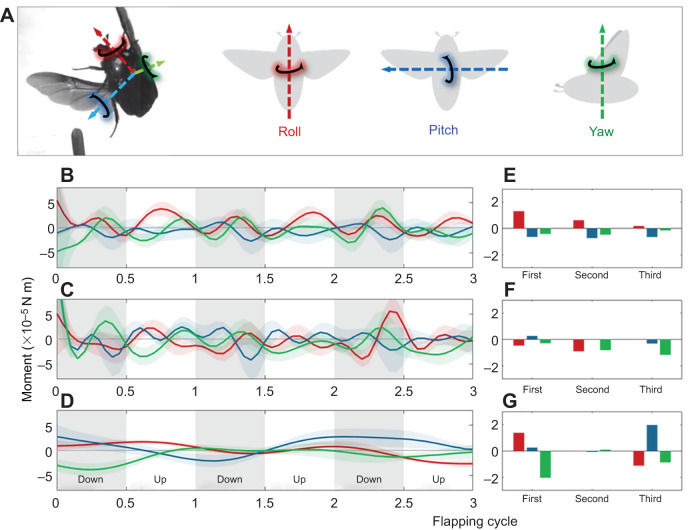
**Force moments.** (A) Illustration of the body axes. (B,C,E,F) instantaneous (B,C) and cycle mean (E,F) net-torque (*M*_aero_) from flapping flexible (B,E) and rigid (C,F) wings. (D,G) instantaneous (D) and cycle mean (G) torque (*M*_actual_) required to rotate the body in the observed rotation. The instantaneous torque panels show means±s.e.m. (*n*=23). Grey areas denote the downstroke phase.

In 20 of the beetles, for which we had body mass data, the average vertical force calculated from the quasi-steady model succeeded in explaining 75±7% of body weight support in air (after accounting for any vertical acceleration of the beetle). The beetle's aerial rotation involved mostly roll and yaw (Figs 2A and 3E). Concordantly, the roll torque estimated from the wing kinematics peaked at mid-upstroke and generated rotation towards the inner wing in the first two flapping cycles (Fig. 7B; see Fig. S3 for the contribution of each wing). Yaw torques also peaked at mid-stroke and on average generated rotation towards the outer wing. Applying the incidence angle of the rigid leading edge in the model gave a net roll torque which would not explain the observed body rotation (Fig. 7B; Fig. S3). The estimation of the torque required to rotate the beetles about their body axes was in general agreement with the net aerodynamic torque due to flapping the flexible wings (Fig. 7E,G). The agreement was most pronounced in the first flapping cycle and decreased in the consequent cycles. A breakdown of the model to show the contribution of each of the forces to the net torque can be found in Figs S4 and S5, and Table S2.

## DISCUSSION

The empirical measurements reported here demonstrate that temporal asymmetry in wing deformation between contralateral wings is a significant component of the flapping asymmetry during rose chafer turns. Namely, we show that, in rose chafers, the asymmetry in flapping kinematics of the rigid section of the wing (leading edge) is mitigated by chord-wise wing compliance, resulting in smaller asymmetry in the incidence angle of contralateral wings.

### Steering wing kinematics

To rotate their body about the vertical axis, rose chafers increased the flapping amplitude of the inner wing and increased and decreased the incidence of this wing, compared with the contralateral (outer) wing, at the downstroke and upstroke, respectively (Fig. 3). The attenuation in contralateral differences in flapping kinematics during the three flapping cycles observed in the means of all beetles (Fig. 3) probably reflects higher torques applied at the beginning of the turn. However, it may also have been affected by our combining data from beetles performing slower or larger turns, which require asymmetric flapping during three flapping cycles, with data from beetles performing fast or smaller turns, which require asymmetric flapping during two or one flapping cycles. There seems to be some evidence for a change in directionality of the flapping asymmetry towards the last upstroke (flapping cycle 3 in Fig. 3), supporting the idea that counteracting torques at the end of the manoeuvre actively stop the rotation of the body (Cheng et al., 2011; Hedrick et al., 2009).

Studies on fruit flies described their steering kinematics for yaw and roll during free flight (Beatus and Cohen, 2015; Bergou et al., 2010). To yaw, fruit flies modulate their wing pitch. They increase the pitch angle of the downstroke compared with the upstroke in the inner wing (Bergou et al., 2010). Our beetle did the same, as seen in the first two flapping cycles of Fig. 6B. To roll, fruit flies increased the amplitude of the outer wing (Beatus et al., 2015), which contradicts our observations on the beetles. However, the fruit flies also supinated the outer wing at an angle that was smaller relative to that for the inner wing. As a result, the AoA of the outer wing should be higher than that of the inner wing, at least during part of the upstroke. This was the case in our beetles as well (Fig. 6B). The steering kinematics of our beetles seem to reflect the fact that the manoeuvres were not about one primary body axis but rather involved a combination of roll, pitch and yaw.

### Wing deformation

The extensive wing deformations during both the downstroke and upstroke followed the general trend previously reported for these beetles during straight slow flight (Meresman and Ribak, 2017). However, while turning, the flapping asymmetry resulted in differences in the chord-wise deformation of contralateral wings that peaked at stroke reversals and mid-strokes (mostly upstrokes) (Fig. 5). The high asymmetry in elastic deformation during stroke reversal was primarily due to slight changes in timing of the left and right wing rotation (see below). In contrast, the asymmetry observed in the angle of incidence (α­_f_) at midstroke was not a temporal effect of one wing lagging behind the other. Rather, it seemed to be a genuine change in wing deformation due to asymmetry in force production.

During stroke reversals, the rapid wing rotation (pitch) typically generates suction above the wings, eliciting aerodynamic forces when the wings are at their slowest translation speed (Dickinson et al., 1999). The wing rotation generates a wave-like motion running from the wing tip towards the wing base through the flexible trailing edge (Movie 1). In addition to the flexibility of the wing veins and membrane themselves, flower chafer wings have resilin (a rubber-like protein) in the connection between some of the wing veins, further contributing to elastic wing deformation during flight (Haas et al., 2000). The resulting twist causes the proximal area to become highly cambered. Flexibility of the proximal trailing edge, as well as the different timing of wing rotation between contralateral wings, can affect steering by creating instantaneous asymmetry in force production during stroke reversal. Dickinson et al. (1993) showed that tethered fruit flies actively adjust the wing rotation delay between contralateral wings during visually evoked attempted yaw turns. As a result, the outer wing of the tethered fruit flies rotates in advance of the inner wing and the advanced timing is correlated with increased flapping amplitude of the same wing. We found the opposite in our free-flying beetles. The inner wing had a larger flapping amplitude and it consistently started rotating in advance of the outer wing during stroke reversals (Fig. 6). The discrepancy may reflect tethering effects and/or the fact that our manoeuvres were not pure yaw rotations. Regardless of the discrepancy, a closer examination of the rotation of the rigid leading edge of both wings in our beetles (Fig. 6A) reveals that they rotated at the same time but that the rotation amplitude (total angular displacement about the wing length) of the outer wing was 26% higher, meaning that it was moving at a higher mean angular velocity. The compliant area of the trailing edge lagged behind the leading edge, thus delaying the rotation of both wings (compare the slopes adjacent to the stroke reversal points in Fig. 6), but less so for the inner wing. Thus, the difference in stroke reversal timing between contralateral wings might simply be a consequence of contralateral force asymmetry acting on chord-wise flexible wings. Chord-wise flexibility also attenuated the differences in wing rotation amplitude, allowing a smoother transition between the two half-strokes and starting each half-stroke with a larger AoA in both wings.

During wing translation, the chord-wise wing deformation resulted in twist and camber, thus increasing the effective AoA beyond the incidence angle of the rigid leading edge in both the inner and outer wings, and decreasing the contralateral asymmetry in incidence angle of the compliant wings compared with the rigid leading edges (Figs 3B and 6A). Nevertheless, the incidence angle in the inner wing remained larger during the downstroke and lower during the upstroke compared with that for the outer wing. During upstroke, the incidence angle on the compliant inner wing was up to 12±2% smaller than on the outer wing. As explained above, lowering the pitch angle of the inner wing should affect both drag and lift of this wing, prompting an unbalanced force couple that rotates the beetle in the turning direction (Bergou et al., 2010; Ribak et al., 2012).

Indeed, the flexible wings generated torques that rotated the beetles in the direction towards the inner wing (Fig. 7). Our model underestimated forces, as evident by the mean vertical force explaining only 75% of the body weight of the beetles. Such underestimation could have emerged from a combination of several simplifying assumptions made by the model. However, we were interested in the asymmetry in force production between the inner and outer wings rather than the magnitude of these forces. In that respect, it is important to note that the same assumptions regarding force production were applied in the calculation of force and torques from both wings. With respect to torques, the moment arm in our simplified aerodynamic torque model was always based on point P2 on the wing. However, we note that during flapping the span-wise position of the resultant aerodynamic force may shift, giving flying animals another way to control asymmetry in torque production between contralateral wings.

The increased incidence angle of the flexible wing (α_f_) compared with the rigid wing (α_r_), resulting in the former producing larger torques, implies that chord-wise wing flexibility contributes to increasing the torque generated by each wing, so that a small difference in the incidence angle between contralateral wings provides sufficient net torque to rotate the body with higher angular accelerations (Fig. 7). In contrast, the rigid wing kinematics results in odd torques simply because the flapping kinematics of the leading edge is intended to actuate the flexible wing so that, upon deforming, it will assume the desired incidence angle.

Two important aspects of flight performance are the ability to hover and to manoeuvre. While the former requires the ability to hold position steadily in the air, the latter requires rapid deviation from the steady-state flight. Consequently, flight stability and manoeuvrability tend to result in a trade-off, as a design for high flight stability resists manoeuvring, whereas high manoeuvrability requires constant corrections to maintain stable flight (Dickinson et al., 1998; Ellington, 1991). Zeyghami et al. (2019) used computational fluid dynamic modelling to analyse the general case of turn termination using flexible flapping wings. They show that when the body is rotating about an axis normal to the stroke plane, elastic contralateral wings actuated by their leading edges using the same flapping kinematics operate a bilateral asymmetry that results from unequal passive wing pitch. The resulting torques contribute to higher manoeuvrability by acting against the stabilizing flapping counter-torque (Hedrick et al., 2009) that occurs on rigid wings and works to stop body rotation.

In the more complex turns of rose chafers, the fact that wing compliance increases the torques generated by each wing adds the potential for higher manoeuvrability because these torques are used to rotate the body in air. However, our findings suggest that wing compliance reduces steering asymmetry in wing pitch, at least during turn initiation. It can thus improve flight stability by providing a dampened steering response and attenuating undesirable asymmetries due to flight perturbations. We suggest that such dampening, due to wing compliance, can provide more control for precise low-speed manoeuvring while flying among vegetation and landing on flowers. It can also assist in responding to external perturbations such as turbulence (Mistick et al., 2016) when performing these delicate landing manoeuvres in the presence of wind.

While we used empirical methods to demonstrate the effect of compliant wings on low-speed flight and manoeuvring, the complex fluid–structure interaction of compliant flapping wings during manoeuvring warrants further exploration using more advanced computational fluid dynamic modelling. Nonetheless, the empirical findings presented here emphasize the need to take into account the contralateral differences in elastic wing deformation when analysing the flight dynamics of insects in general, and manoeuvring flight in particular.

## Supplementary Material

Supplementary information
