# Supplementary material for: Elastic wing deformations mitigate flapping asymmetry during manoeuvres in rose chafers (Protaetia cuprea)
Source: J Exp Biol. 2020 Dec 22;223(24):jeb225599. doi: 10.1242/jeb.225599 (PMC7774887; doi:10.1242/jeb.225599)
Supplement: Supplementary information [file jexbio-223-225599-s1.pdf]

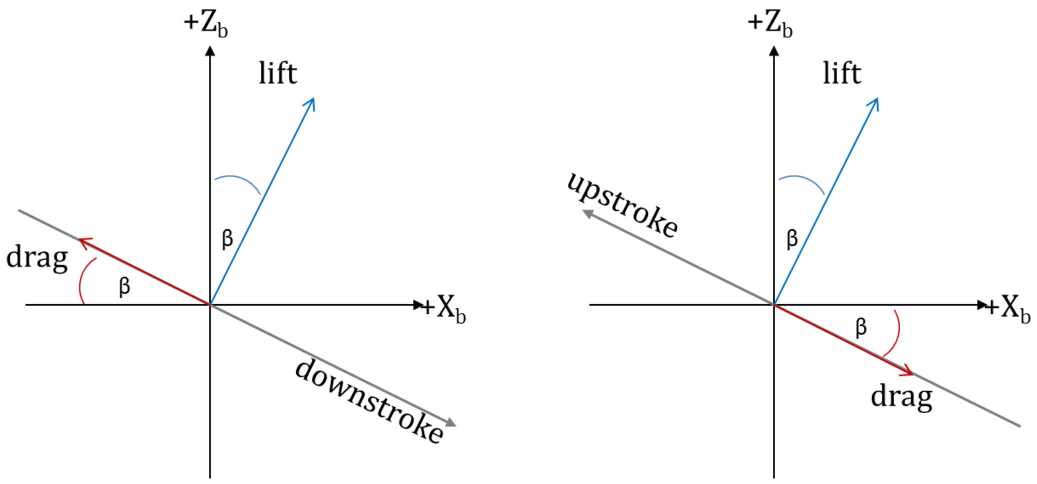

Fig. S1. Drag and lift vectors (blue and red, respectively) and the stroke plane angles (β) used for the calculation of equations (Eq. 21 – 22 in the main text). X_b and Z_b are the longitudinal and dorso-ventral body axes. Arrowheads of these axes point to (positive values) the anterior and dorsal sides of the beetle, respectively). Grey arrows illustrate the stroke plane in the sagittal (XZ) plane and point in the direction of the wing movement.

Journal of Experimental Biology • Supplementary information

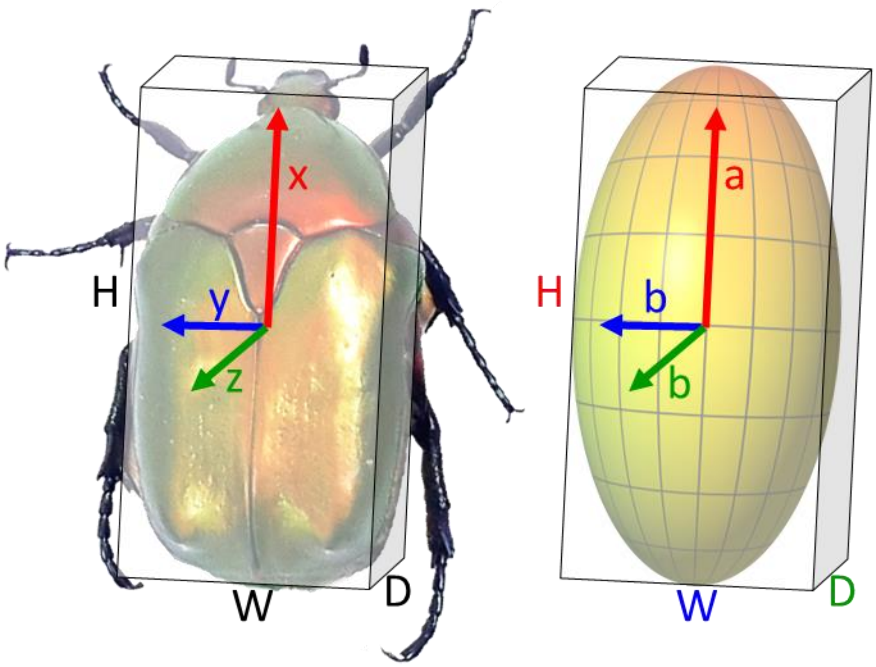

Fig. S2. Body parameters of *P. cuprea* and axes of a matching prolate spheroid. x, y, and z correspond to the beetles' roll, pitch, and yaw axes, respectively.

Journal of Experimental Biology • Supplementary information

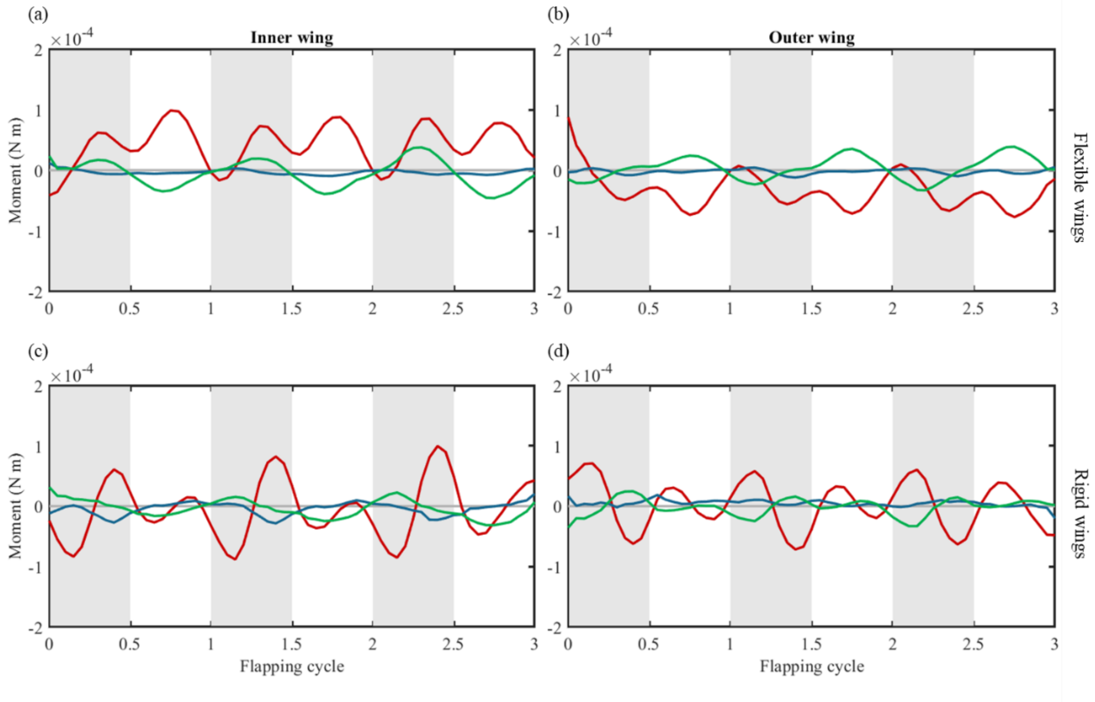

Fig. S3. Mean (n=23) force moments calculated from the flexible (*a*-*b*) and rigid (*c*-*d*) parts of the outer and inner wing. The torques are shown about the longitudinal (roll, *x*, red), lateral (pitch, *y*, blue) and dorso-ventral (yaw, *z*, green) axes. Grey background denotes the downstrokes.

Journal of Experimental Biology • Supplementary information

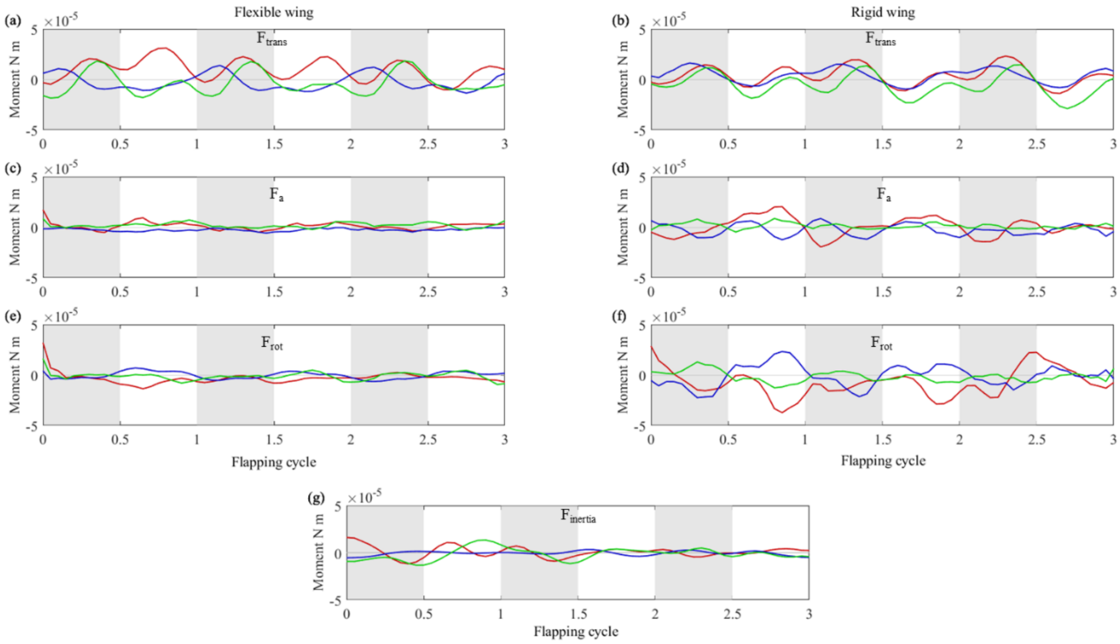

Fig. S4. Time-varying moments. The contribution of the various force components in the model to the total net torque (inner wing+outer wing) generated for rotating the beetle: F_{trans} (a-b), F_a (c-d), F_{rot} (e-f) and $F_{inertia}$ (g). The calculations are shown once using the AoA of a flexible wing (a,c,e) and once for a rigid wing (b,d,f). $F_{inertia}$ is the same for both the flexible and rigid wings. Red, blue and green lines denote the net-moment about the beetles' roll, pitch and yaw axes, respectively. Grey, and white backgrounds denote down- and upstrokes, respectively.

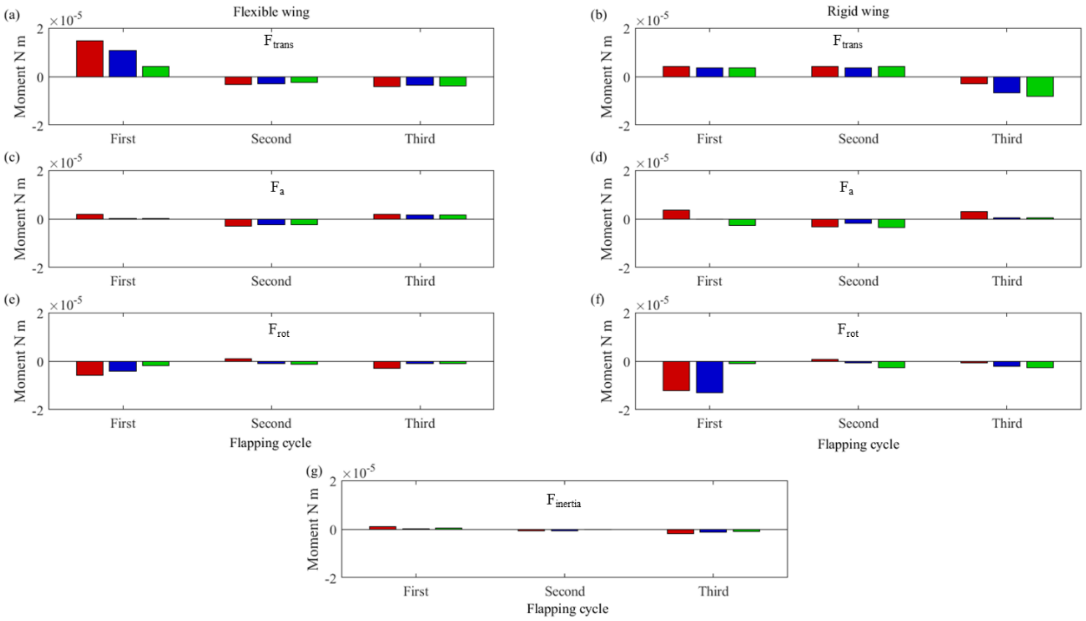

Fig. S5. Mean moments per flapping cycle. Same as in Figure S4 but for the mean torque for each of the flapping cycles 1-3.

Table S1. Supporting statistics for the instantaneous angles comparisons. Two-tailed, paired-sample t-tests (df = 22) were carried out in order to test for difference in instantaneous kinematic angles between the inner and outer wings throughout three flapping cycles. P values <0.05 are in bold font.

Flapping	Flapping angle				Incidence angle				Deviation angle			
Time	Inner	Outer	t value	p value	Inner	Outer	t value	p value	Inner	Outer	t value	p value
0.00	146.089	140.826	1.230	0.232	110.55	150.66	-2.112	**0.046**	1.483	6.398	-0.844	0.408
0.05	152.677	148.086	1.413	0.172	94.16	100.56	-1.237	0.229	8.317	2.756	4.083	**<0.001**
0.10	144.777	144.221	0.202	0.842	54.11	61.46	-1.627	0.118	7.551	2.765	3.241	**0.004**
0.15	131.603	134.095	-1.004	0.326	40.94	32.79	2.258	**0.034**	5.866	-0.767	3.892	**0.001**
0.20	116.865	122.262	-1.938	0.066	49.88	41.66	3.770	**0.001**	5.106	-2.146	4.192	**<0.001**
0.25	100.303	107.733	-2.665	**0.014**	49.79	43.30	4.302	**<0.001**	5.885	-1.022	4.239	**<0.001**
0.30	82.716	91.493	-2.693	**0.013**	43.43	40.02	1.783	0.088	6.117	-0.376	3.783	**0.001**
0.35	65.568	75.033	-2.679	**0.014**	41.90	38.43	2.040	0.054	5.166	-0.069	3.414	**0.002**
0.40	49.970	60.391	-2.731	**0.012**	42.22	41.06	0.589	0.562	4.878	0.308	3.341	**0.003**
0.45	38.433	49.062	-2.818	**0.010**	50.93	47.52	1.974	0.061	4.400	0.446	3.058	**0.006**
0.50	31.881	42.301	-3.193	**0.004**	70.66	63.07	2.338	**0.029**	4.976	0.826	2.927	**0.008**
0.55	30.414	38.837	-3.091	**0.005**	98.53	85.49	3.516	**0.002**	6.877	0.427	4.238	**<0.001**
0.60	36.669	41.017	-2.082	**0.049**	128.43	115.71	2.975	**0.007**	6.935	1.001	4.300	**<0.001**
0.65	49.244	50.826	-0.748	0.463	136.10	139.09	-1.167	0.256	3.826	-2.190	3.894	**0.001**
0.70	63.139	63.823	-0.302	0.766	130.49	134.13	-1.171	0.254	2.522	-5.152	5.358	**<0.001**
0.75	78.594	77.755	0.333	0.743	128.39	123.77	1.975	0.061	3.501	-4.578	5.546	**<0.001**
0.80	95.976	92.627	1.205	0.241	140.58	122.64	7.183	**<0.001**	4.172	-1.952	3.978	**0.001**
0.85	113.808	109.209	1.481	0.153	149.59	130.49	6.141	**<0.001**	4.219	0.303	2.866	**0.009**
0.90	130.399	125.052	1.727	0.098	148.46	135.59	3.838	**0.001**	4.995	2.006	2.127	**0.045**
0.95	143.330	137.003	1.968	0.062	139.15	132.02	2.101	**0.047**	5.318	2.586	1.936	0.066

Journal of Experimental Biology • Supplementary information

1.00	151.081	145.434	1.842	0.079	122.33	120.96	0.346	0.732	6.671	2.270	3.613	**0.002**
1.05	151.676	148.232	1.174	0.253	86.52	91.80	-1.135	0.268	8.757	3.111	4.484	**<0.001**
1.10	143.072	142.215	0.298	0.768	51.42	48.55	0.677	0.505	7.476	2.521	3.557	**0.002**
1.15	130.279	130.549	-0.096	0.924	41.25	33.51	3.302	**0.003**	4.454	-1.320	3.912	**0.001**
1.20	115.427	117.891	-0.856	0.401	49.95	45.84	2.111	**0.046**	4.013	-1.444	3.373	**0.003**
1.25	98.920	101.842	-0.882	0.388	48.35	43.36	3.426	**0.002**	5.152	-0.035	2.997	**0.007**
1.30	80.474	85.576	-1.443	0.163	44.52	38.95	3.288	**0.003**	4.523	1.190	1.895	0.071
1.35	63.287	69.066	-1.559	0.133	42.02	39.82	1.606	0.123	4.232	1.876	1.922	0.068
1.40	48.446	54.697	-1.690	0.105	44.65	41.34	1.966	0.062	3.941	1.756	1.787	0.088
1.45	38.098	44.331	-1.776	0.090	54.17	50.16	2.066	0.051	3.396	1.213	1.885	0.073
1.50	31.972	38.594	-1.959	0.063	73.84	69.36	1.314	0.202	4.142	0.842	2.646	**0.015**
1.55	31.480	36.451	-1.683	0.107	105.16	92.07	2.972	**0.007**	5.906	1.564	3.265	**0.004**
1.60	39.014	41.456	-1.037	0.311	133.18	123.68	2.296	**0.032**	4.323	1.425	2.135	**0.044**
1.65	50.786	53.325	-1.319	0.201	134.09	137.98	-1.435	0.165	1.638	-2.429	2.721	**0.012**
1.70	64.258	67.004	-1.437	0.165	125.79	129.75	-1.534	0.139	1.045	-4.846	3.426	**0.002**
1.75	79.044	82.330	-1.874	0.074	127.96	124.92	1.231	0.231	2.777	-3.164	3.518	**0.002**
1.80	96.412	98.336	-1.008	0.324	138.28	125.41	4.173	**<0.001**	4.304	-0.144	2.295	**0.032**
1.85	113.162	116.236	-1.663	0.111	146.62	137.36	2.996	**0.007**	3.885	0.676	1.748	0.094
1.90	129.018	131.826	-1.368	0.185	146.43	140.13	1.589	0.126	3.834	1.935	1.200	0.243
1.95	141.262	143.221	-0.844	0.408	138.44	133.42	1.984	0.060	3.975	2.250	1.229	0.232
2.00	148.913	150.156	-0.543	0.593	119.85	116.23	1.033	0.313	5.176	3.227	1.476	0.154
2.05	148.981	149.469	-0.208	0.837	83.31	78.08	1.121	0.274	6.963	5.247	1.143	0.265
2.10	140.061	140.954	-0.371	0.714	48.08	43.58	1.526	0.141	5.763	2.655	1.878	0.074
2.15	127.319	128.327	-0.454	0.654	39.11	37.74	0.503	0.620	3.122	0.382	1.559	0.133
2.20	112.612	113.837	-0.509	0.616	48.38	49.23	-0.383	0.705	2.530	1.183	0.717	0.481
2.25	96.435	96.588	-0.053	0.958	46.48	42.80	2.016	0.056	2.738	2.523	0.122	0.904
2.30	78.670	78.906	-0.068	0.946	43.42	38.98	2.501	**0.020**	2.887	3.301	-0.234	0.817
2.35	63.056	61.349	0.441	0.663	43.84	40.72	2.109	**0.047**	3.139	4.002	-0.545	0.591
2.40	49.176	47.540	0.410	0.686	47.20	41.67	3.608	**0.002**	2.350	3.862	-1.139	0.267

Journal of Experimental Biology • Supplementary information

2.45	39.323	38.734	0.156	0.878	58.30	55.54	0.814	0.424	2.744	4.494	-1.401	0.175
2.50	33.466	34.222	-0.217	0.830	79.42	76.67	0.558	0.583	3.824	4.996	-0.924	0.366
2.55	34.085	34.206	-0.044	0.965	110.11	104.11	1.082	0.291	3.716	4.660	-0.710	0.485
2.60	42.084	41.888	0.084	0.934	132.74	133.23	-0.120	0.906	3.236	3.221	0.012	0.991
2.65	54.019	54.729	-0.309	0.761	132.26	139.98	-2.898	**0.008**	-0.256	-1.606	0.897	0.380
2.70	66.710	70.291	-1.302	0.206	126.92	131.53	-1.862	0.076	0.250	-3.257	1.982	0.060
2.75	81.648	86.006	-1.405	0.174	128.69	126.70	0.588	0.562	1.748	-1.770	2.119	0.046
2.80	98.405	103.166	-1.444	0.163	138.50	132.69	1.468	0.156	3.178	0.021	1.945	0.065
2.85	115.318	121.012	-1.811	0.084	144.86	142.21	0.841	0.409	3.120	1.439	0.909	0.373
2.90	130.675	136.352	-1.950	0.064	144.81	142.33	0.860	0.399	2.240	2.147	0.060	0.953
2.95	141.881	147.115	-1.835	0.080	134.90	130.46	2.241	0.035	2.945	3.802	-0.600	0.554
3.00	148.257	152.823	-1.880	0.073	116.16	108.41	2.544	0.018	3.389	4.206	-0.605	0.551

Journal of Experimental Biology • Supplementary information

Table S2. Mean moments (N m) per flapping cycle.

Component		First		Second		Third	
		Flexible	Rigid	Flexible	Rigid	Flexible	Rigid
F_{trans}	Roll	1.48E-05	4.08E-06	1.08E-05	3.52E-06	4.02E-06	3.47E-06
	Pitch	-3.37E-06	4.22E-06	-2.91E-06	3.52E-06	-2.44E-06	4.03E-06
	Yaw	-4.01E-06	-2.93E-06	-3.57E-06	-6.65E-06	-3.85E-06	-8.24E-06
F_a	Roll	1.80E-06	3.53E-06	9.74E-08	-6.49E-09	1.49E-07	-2.70E-06
	Pitch	-2.89E-06	-3.20E-06	-2.46E-06	-1.82E-06	-2.42E-06	-3.57E-06
	Yaw	1.98E-06	2.89E-06	1.57E-06	5.54E-07	1.47E-06	4.92E-07
F_{rot}	Roll	-5.97E-06	-1.22E-05	-4.15E-06	-1.29E-05	-2.00E-06	-1.10E-06
	Pitch	9.52E-07	7.35E-07	-9.59E-07	-8.45E-07	-1.38E-06	-2.67E-06
	Yaw	-2.93E-06	-6.96E-07	-8.70E-07	-2.28E-06	-1.03E-06	-2.63E-06
$F_{inertia}$	Roll	8.85E-07		1.47E-07		5.26E-07	
	Pitch	-6.70E-07		-5.79E-07		-2.24E-07	
	Yaw	-1.81E-06		-1.25E-06		-1.03E-06	

Journal of Experimental Biology • Supplementary information

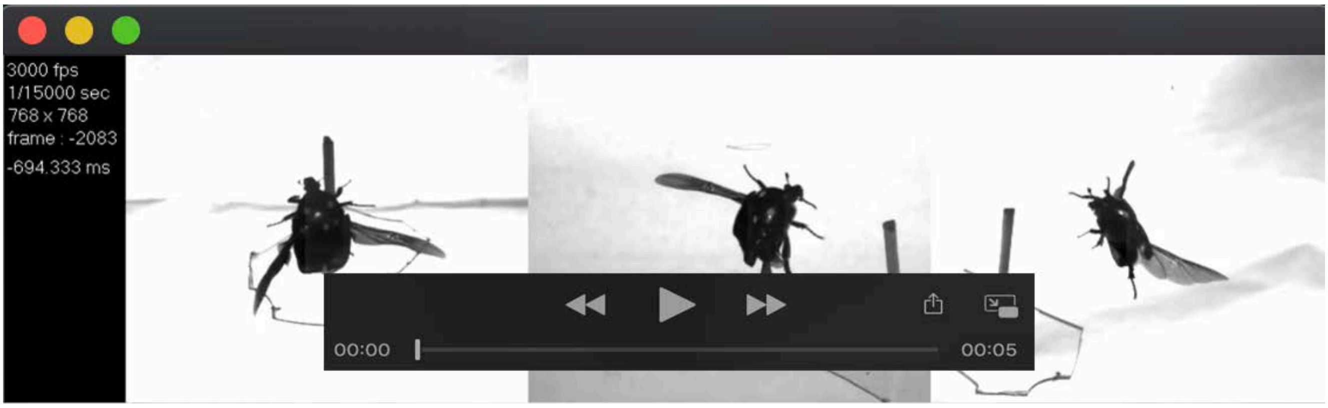

Movie 1. A high-speed film of a flower chafer, *P. cuprea*, performing aerial rotation during low-speed free flight.

Journal of Experimental Biology • Supplementary information
